# Autonomous planning and control of soft untethered grippers in unstructured environments

**DOI:** 10.1007/s12213-016-0091-1

**Published:** 2016-09-12

**Authors:** Federico Ongaro, Stefano Scheggi, ChangKyu Yoon, Frank van den Brink, Seung Hyun Oh, David H. Gracias, Sarthak Misra

**Affiliations:** 1 Surgical Robotics Laboratory, University of Twente, Enschede, 7522 NB The Netherlands; 2 The Johns Hopkins University, Baltimore, MD 21218 USA; 3 University of Groningen and University Medical Centre Groningen, Groningen, 9713 GZ The Netherlands

**Keywords:** Soft robotics, Gripper, Self-folding, Biocompatible, Autonomous

## Abstract

The use of small, maneuverable, untethered and reconfigurable robots could provide numerous advantages in various micromanipulation tasks. Examples include microassembly, pick-and-place of fragile micro-objects for lab-on-a-chip applications, assisted hatching for *in-vitro* fertilization and minimally invasive surgery. This study assesses the potential of soft untethered magnetic grippers as alternatives or complements to conventional tethered or rigid micromanipulators. We demonstrate closed-loop control of untethered grippers and automated pick-and-place of biological material on porcine tissue in an unstructured environment. We also demonstrate the ability of the soft grippers to recognize and sort non-biological micro-scale objects. The fully autonomous nature of the experiments is made possible by the integration of planning and decision-making algorithms, as well as by closed-loop temperature and electromagnetic motion control. The grippers are capable of completing pick-and-place tasks of biological material at an average velocity of 1.8 ±0.71 mm/s and a drop-off error of 0.62 ±0.22 mm. Color-sensitive sorting of three micro-scale objects is completed at a velocity of 1.21 ±0.68 mm/s and a drop-off error of 0.85 ±0.41 mm. Our findings suggest that improved autonomous untethered grippers could augment the capabilities of current soft-robotic instruments especially in advancedtasks involving manipulation.

## Introduction

Small-scale robots have shown promising results in a broad variety of tasks, ranging from micromanipulation [1–4] and microassembly [5, 6] to minimally invasive surgical (MIS) interventions, such as biopsies [7] and targeted drug delivery [8–11]. In many of these tasks there is a need to move and transport objects with high precision in unstructured environments. Examples include pick-and-place of fragile objects for lab-on-a-chip applications, assisted hatching for *in-vitro* fertilization, assembly and repair of printed circuit boards (PCB) and of microelectromechanical systems (MEMS), angioplasty and embolization [12–16]. Untethered small-scale robots with soft, maneuverable and reconfigurable components could perform these procedures, improving functionality. For this purpose, the robots would have to possess reliable and robust motion and grasping capabilities. Soft grippers might represent a viable solution. The reliability and robustness of large-scale soft grippers have been heavily demonstrated, as they are a reality in the market for manipulators [17–19]. Additionally, closed-loop control would be necessary to achieve the required high levels of precision and robustness.

However, the implementation of closed-loop control at such scales presents significant challenges. Furthermore, small-scale robots have to satisfy at least four requirements for applicability. They must: (1) possess an untethered structure that allows them to move in tortuous environments; (2) allow manipulation along accurate trajectories in unstructured environments; (3) have sizes that allow them to reach and move within constrained environments; and (4) be capable of detecting and promptly reacting to variations of their surroundings. Various untethered agents capable of manipulation on small size scales have been described previously [20–23]. Among these, self-folding hydrogel soft grippers allow the decoupled control of motion and grasp [24]. Their ability to wirelessly harness power from the surrounding environment offers a solution to requirement #(1) [7, 25]. They can be fabricated in sizes that are compatible with applications such as *in-vitro* fertilization, PCB and MEMS assembly, and allow access to the major vascular vessels, satisfying requirement #(3) [26–28]. Furthermore, requirements #(2) and #(4) can be fulfilled by using accurate decision-making and tracking algorithms, closed-loop control, and fast online trajectory planning that exploits the ferromagnetic properties of the grippers. The synergy of such algorithms can make the system fully-autonomous, greatly increasing performance consistency and success rates [29].

Although several pathfinding algorithms have been developed in recent years, only few have been applied to magnetic agents in an experimental scenario. Khalil et al. implemented a 2D path planner based on the A* algorithm and the Artificial Potential Field approach for paramagnetic microparticles [30]. A path planner based on the Rapidly-exploring Random Tree algorithm was developed for biological cell transportation using optical tweezers in [31]. Recently, an experimental comparison of six path planning algorithms when applied to the motion control of paramagnetic microparticles was presented in [32].

**Fig. 1:**
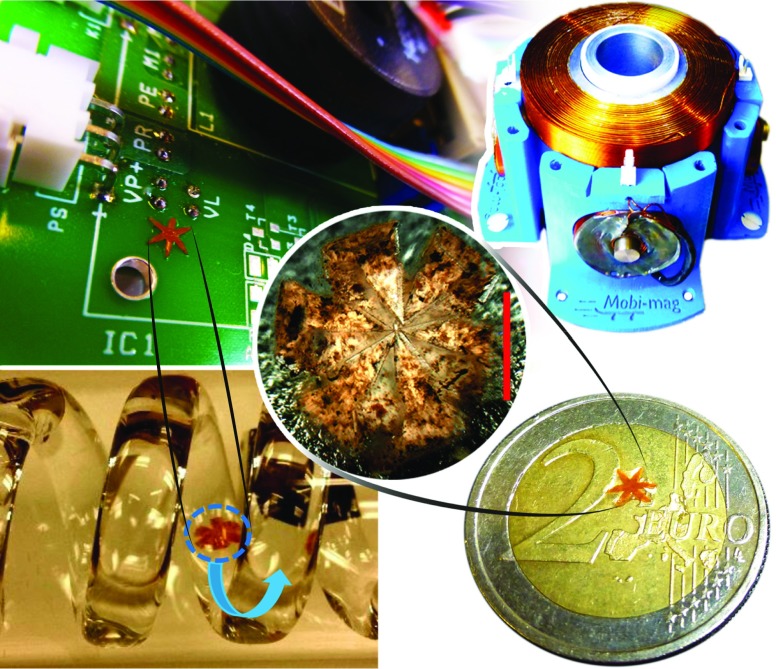
*Top Left:* Image of a soft untethered gripper on a printed circuit board. *Top Right:* The electromagnetic setup consisting of six orthogonally-oriented electromagnetic coils. The electromagnetic coils have a cutoff frequency of 70 Hz and a maximum current rating of 1 A. The system generates a maximum magnetic field and magnetic field gradient of 15 mT and 60 mT/m, respectively. *Center:* Image of the closed gripper. *Bottom Left:* Video snapshot images of the gripper navigating through a tube with an inner diameter of 3.2 mm, a size smaller than most of the major vessels in the human body. *Bottom Right:* Image of the gripper on a 2 *€* coin. The scale bar is 0.4 mm

In this work, we show the potential of soft, untethered grippers in tasks that involve autonomous manipulation, as well as obstacle recognition and manipulation in unstructured environments (Fig. 1). Two experiments are performed for this purpose. In the first, soft bilayer grippers are used to perform the pick-and-place of soft irregularly-shaped biological material in the presence of both static and dynamic obstacles. The second experiment demonstrates that soft grippers can autonomously recognize, classify, and manipulate regularly-shaped micro-scale rigid objects. In both experiments, the soft grippers utilize their six tips to grasp a micro-scale object and constrain it in all directions. (Fig. 1).

It should be noted that soft-grippers were previously manipulated using robotic control [22]. However, there are several important differences between our work and this previously reported study. Firstly, previously utilized grippers had a homogeneous mechanical composition, whereas our soft grippers are designed with rigid segments and flexible joints. This design feature seeks to mimic biological appendages such as hands, and ensures a robust grasp of the transported microscale objects, even in the case of abrupt jerks. Secondly, as compared to previous work in which the gripper itself was not magnetic but rather was moved by a magnetic object, we have succeeded in doping the grippers themselves with magnetic nanoparticles to allow motion control even when the grippers are not grasping a magnetic object. Furthermore, bulk magnetic doping was chosen instead of a surface coating to preserve the soft material characteristics of the gripper and prevent the corrosion or delamination of the magnetic cladding in media such as acid fluids or blood. Additionally, in this work the grippers are fully autonomous; i.e., they are able to interact with the environment and automatically adapt their behavior in real-time to changes in the scenario. Consequently, the aforementioned tasks can be autonomously performed in a faster, safer, and more robust way. Finally, a novel high sampling rate technique that makes use of an XYZ Cartesian robot is used in order to model and validate the electromagnetic system. Such validation at high density and resolution not only ensures the reliability of the model, but also enables the reliable computation of the magnetic field gradient.

The rest of the paper is organized as follows. Section 2 describes the fabrication of the soft hydrogel grippers. The techniques used to track and detect the grippers are reviewed in Section 3. Section 4 presents the motion planner used to move them along obstacle-free paths, here also the modeling of the electromagnetic setup and its validation are described. The experimental validation is presented in Section 5. Finally, Section 6 summarizes the main contributions of this paper, and proposes possible avenues for future research.

## Fabrication of the hydrogel grippers

**Fig. 2:**
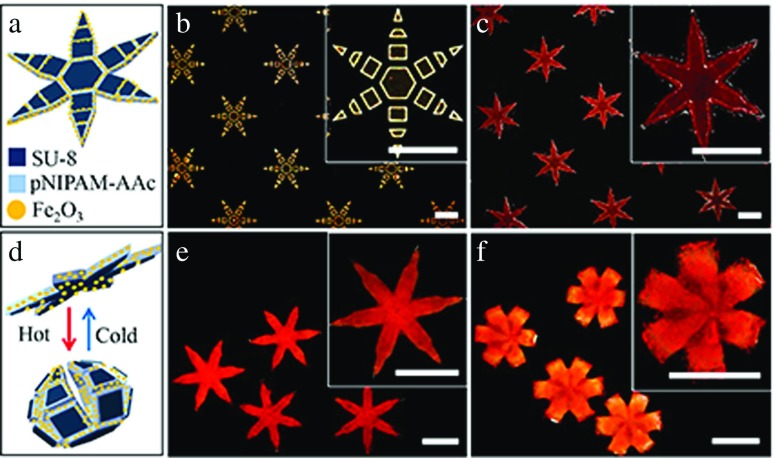
Schematic and experimental images of the fabrication and operation of the soft bilayer grippers. **a** Schematic showing the bilayer composed of a stiff, segmented, SU-8 polymer coupled with a 5 % (w/w) magnetic nanoparticle (Fe _2_ *O*_3_) doped thermally stimuli responsive poly(N-isopropylacrylamide-co-acrylic acid) (pNIPAM-AAc) layer (**b–c**). Experimental images after wafer scale patterning of the two layers. **d** Schematic and (**e–f**) experimental images showing operation of the grippers with reconfiguration from, **e** a flat state at room temperature to, **f** a closed state in which the temperature is raised to 25.3 ^∘^C

The detailed gripper fabrication process has been described previously [28] with one design change in that we replaced the stiff polymer with SU-8, a widely available photopatternable polymer. Briefly, bilayer grippers (Fig. 2a) were composed of lithographically photopatterned segmented SU-8 (Fig. 2b) and continuous poly (N-isopropylacrylamide-co-acrylic acid) (pNIPAM-AAc) (Fig. 2c). Furthermore, in order to make magnetically responsive soft grippers, 5 % (w/w) biocompatible iron (III) oxide (Fe _2_ *O*_3_, Sigma-Aldrich, St. Louis, USA) 50 nm nanoparticle powder was mixed with the pNIPAM-AAc solution prior to crosslinking. The soft grippers open and close reversibly due to a phase transition and associated swelling or shrinkage in the pNIPAM-AAc layer in response to temperature changes (Fig. 2d–f). When fully opened, the grippers have an hexagram shape with a tip-to-tip distance of 4 mm. When fully closed, the grippers have the shape of a sphere with a 0.4 mm radius.

The F _2_ *O*_3_ nanoparticle patterning and the magnetic field gradient generated by an array of six orthogonal iron-core electromagnets (Fig. 1) can be used for closed-loop motion control of the grippers. To perform autonomous, accurate and robust pick-and-place of biological material in unstructured environments, the following modules are necessary: (1) an accurate image-guided tracking algorithm; (2) a reactive motion planner which uses the information from the tracker to compute suitable motion control inputs for the gripper. In the next section, we describe the tracking algorithm used to estimate the pose of the gripper.

## Detection and tracking of the grippers

Let $\mathbf {p} = [x,\; y]^{T} \in \mathbb {R}^{2 \times 1}$ be the position of a gripper in 2D space. The state of the gripper is defined as **x**=[*x*, *y*, *v*_*x*_, *v*_*y*_]^*T*^ $\in \mathbb {R}^{4 \times 1}$ where *v*_*x*_, *v*_*y*_ represent its velocities. Let us consider the miniaturized gripper as a second order system controlled by applying suitable force inputs.

**Fig. 3:**
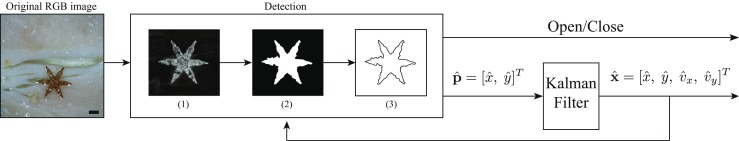
Flowchart showing the tracking process: From left to right, a Red-Green-Blue (RGB) image is taken from the microscope. In the detection phase, the image is converted to Hue-Saturation-Value (HSV) color space. (1) Bounds on the coordinates of the selected color space are employed in order to accurately detect the gripper. (2) Adaptive threshold, morphological filtering, and opening are used. Then, the adjacent blobs are merged and kept for further consideration. (3) A Discrete Fourier Transform is applied on the contour of the segmented area in order to detect the pose $\hat {\mathbf {p}} = [\hat {x},\; \hat {y}]^{T}$ and the configuration (open/close) of the gripper. Finally, a Kalman filter is used to estimate the state $\hat {\mathbf {x}}$ of the gripper, where $\hat {v}_{x}$, $\hat {v}_{y}$ represent the estimated velocities of the agent. The estimated state of the gripper is used in the next frame to speed up the detection procedure. The scale bar is 1 mm

The tracking algorithm is shown in Fig. 3 and estimates the state $\hat {\mathbf {x}}$ and the configuration (open/close) of the gripper at runtime. In the initial phase, it determines the position $\hat {\mathbf {p}}$ of the gripper. This phase is composed of two steps. The first step extracts the contour of the gripper from the binarized color-saturation level of the image. The input to the proposed method is a 1024 ×1024 Red-Green-Blue (RGB) color image acquired using a Charge-Coupled Device (CCD)connected to a microscopic lens. In the presented work, the Hue-Saturation-Value (HSV) color space is selected. Then, the coordinates of the selected color space are bounded; i.e., specific values of the axis of the color space are selected in order to detect a particular color. The bounds are chosen empirically; i.e., by examining the distribution of colors in a preselected set of images. Morphological filtering and opening are used in order to segment the image. Finally, small blobs are removed and the remaining, adjacent blobs are merged and kept for further consideration.

In the second step, normalized Fourier Descriptors (FDs) $Z_{k}, k \in [-15,\dots ,16]$ are obtained by applying the one-dimensional Discrete Fourier Transform (DFT) to the complex-valued representation of the 2D contour points. FDs provide us with the position and scale of the gripper [33]. To increase noise rejection, we ignore all the contours that are not 6-fold symmetric by only tracking objects that satisfy the following conditions:

$$Z_{-5}>\frac{2}{N-2}\left( {\sum}_{k=-15}^{16} Z_{k}-Z_{1} \right), Z_{7}>\frac{2}{N-2}\left( {\sum}_{k=-15}^{16} Z_{k}-Z_{1}\right). $$

Furthermore, we set a threshold constraint on the ratio between the number of pixels of the perimeter and of the area of the contour. This value must be between 0.5 and 1 in order to avoid erroneous tracking of other 6-fold symmetric objects.

To compute the estimated state $\hat {\mathbf {x}}$ from position measurements $\hat {\mathbf {p}}$, we implement a standard Kalman filter [34] to consider the second order model of the gripper and control-inputs. Assuming a constant sampling time Δ*t* of the system, the Kalman filter provides an estimation of the current state $\hat {\mathbf {x}}$ as well as a one-step ahead prediction of $\hat {\mathbf {x}}$. The process and measurement noises are obtained from zero mean multivariate Gaussian distributions *N*(0,**Q**) and *N*(0,**R**), respectively, where $\mathbf {Q}\in \mathbb {R}^{4 \times 4}$ is the process noise covariance matrix and $\mathbf {R} \in \mathbb {R}^{2 \times 2}$ is the measurement noise covariance matrix. **Q** is determined by empirically tuning its parameters, while **R** is computed by analyzing offline the tracked positions of the gripper using a zero-phase filter.

To speed up the detection procedure, temporal continuity is exploited to track the grippers in a sequence of frames. Given the estimated state $\hat {\mathbf {x}}$ of the tracked gripper from the previous frame, the image pixels that are within a preset range from that estimation are kept, whereas the remaining pixels are discarded. The proposed tracker runs at an average frame rate of 50 frame per second on a PC that has an Intel Xeon CPU 3.2 GHz processor and 8 GB of RAM.

## Motion planning and control

The estimated state $\hat {\mathbf {x}}$ of the gripper is then provided to a motion planner for the computation of a collision-free trajectory. Among possible motion planners, a Rapidly-exploring Random Tree (RRT) is used. The RRT outputs forces that are mapped to currents at the electromagnets. For this purpose, a force-current map is developed using Finite Element Model (FEM) analysis.

### Motion planning

The RRT is able to deal with real-valued spaces of extremely high dimension while handling the dynamics of the system. The planner is rooted at the gripper’s initial state $\mathbf {x}_{start} = \hat {\mathbf {x}}$. At each iteration, the algorithm samples a collision-free state **x**_*sample*_ in the environment, finds its nearest neighbors **x**_*nn*_ in the tree, and computes feasible control forces ($\mathbf {F}\in \mathbb {R}^{3\times 1}$) that grow **x**_*nn*_ toward **x**_*sample*_. The output of the RRT is a motion plan Γ;

$${\Gamma} = [(\mathbf{x}_{start},\; \mathbf{F}_{start}),\; \dots, (\mathbf{x}_{S},\; \mathbf{F}_{S})], $$

where *S* is the number of steps (Algorithm 1). Each entry of the motion plan Γ represents a control input that is applied to the agent after every time interval Δ*t*. Unlike path planning algorithms, which provide waypoints to the robot, RRTs directly provide feasible control inputs **F**.

**Figure:**
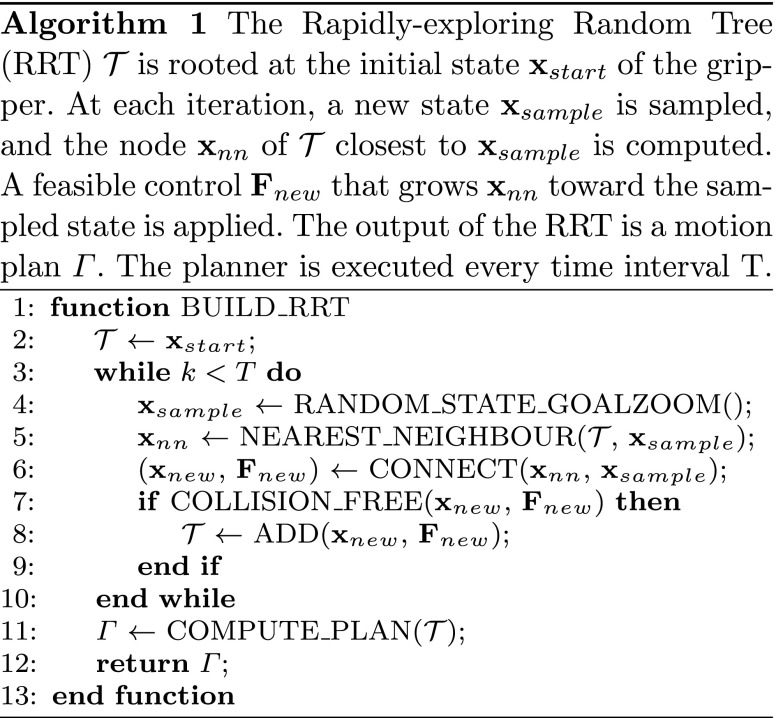

In the experimental validation, an RRT-GoalZoom policy is used to generate a new random state based on a biased coin toss [35]. The algorithm relies on this state to choose a random sample from either a region around the goal or from the whole space. During the planning procedure, instead of extending the RRT by an incremental step, the algorithm iterates until the random state or an obstacle is reached. Finally, a fixed planning time interval of *T*=0.1 s is selected. At the beginning of the task, the RRT computes the motion plan. Successively for each period of duration *T*, the system updates the planned trajectory that will be ready for the next planning time interval *T*. The proposed motion planner allows the system to run autonomously without any need for human intervention during standard operations. This not only reduces the system response time below the average human reaction time (400 ms [36]) to an average of 100 ms, but also lessens – if not eliminates – the need for human supervision. This, combined with the higher repeatability of fully-autonomous systems, greatly increases performance consistency and success rates.

### FEM modeling and validation

Once the RRT planner has computed the obstacle-free motion plan Γ, suitable currents have to be applied to the magnetic coils in order to exert the desired forces $\mathbf {F}_{i}, i = 1,\dots ,S$ on the agent. For this purpose, we use FEM analysis (COMSOL Multiphysics, COMSOL, Stockholm, Sweden) to develop a map between the current at the electromagnets and the magnetic flux density in the workspace. The FEM analysis is successively compared with the measurements of a 3-axis 3MH3 Teslameter (SENIS AG, Baar, Switzerland). To obtain high-density measurements an XYZ Cartesian robot moves the probe through the entire workspace at a velocity of 0.5 mm/s [37, 38]. This technique allows us to compare the results at a resolution of 6.8 ×10^6^ dots/m ^2^ using a total of 1523 samples in the 1.2 ×1.2 cm workspace. The average mismatch and its standard deviation between FEM and measurements – when the electromagnets are fed the maximum current of 1 A – are 7.4 ×10^−3^±2.2 mT and 0.073 ± 0.045 radians for field magnitude and orientation, respectively. The *x*− and *y*−components of the field are then fitted to a quadratic Lowess (LOcally WEighted Scatter plot Smooth) function using MATLAB (MathWorks Inc., Natick, United States). The quality indices of such functions are reported in Table 1. The quadratic Lowess functions are then used to compute the force-current map as described in [39, 40].

**Table 1:** Quality indices for the x- (B _*x*_) and y- (B _*y*_) components of the magnetic field for the obtained fitting function

	SSE (T ^2^)	*R* ^2^	$\bar {R}^{2}$	RMSE (T)
B _*x*_	4.7 ×10^−7^	1.0000	1.000	6.7 ×10^−6^
B _*y*_	2.9 ×10^−7^	1.0000	1.000	5.3 ×10^−6^

The table reports Sum Squared Error (SSE), R-square (*R* ^2^), Adjusted R-square ($\bar {R}^{2}$), and Root Mean Square Error (RMSE)

### Temperature control

The configuration control is provided by a Peltier element. This element controls the temperature of the environment and, consequently, the configuration (open/close) of the gripper. The configuration, determined using FD, is used as feedback for the closed-loop grasp control. In the next section, an experimental validation is performed in order to demonstrate the ability of the grippers to manipulate both biological and non-biological materials in unstructured environments.

## Experimental validation

**Fig. 4:**
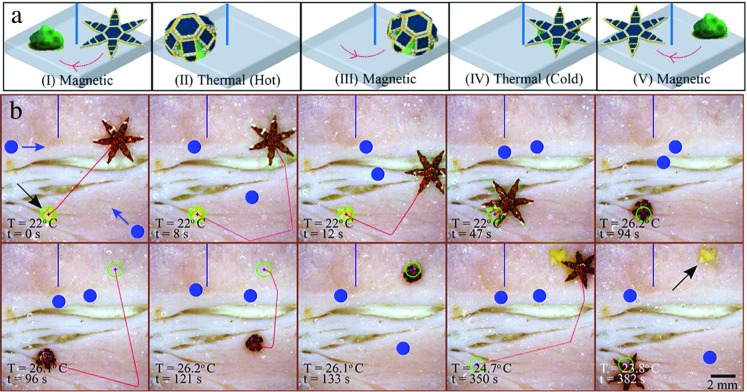
**a** Schematic of a representative obstacle avoidance and biological manipulation experiment. **b** Video snapshots of the gripper during pick-and-place of biological material in an unstructured environment on porcine tissue. The virtual obstacles are shown in blue; the circular ones are dynamic, while the wall on top of the workspace is static. The blue arrows represent the initial motion directions of the dynamic obstacles. The velocity of the horizontally and diagonally moving obstacle is 0.35 mm/s and 0.5 mm/s, respectively. The targeted position is represented the blue cross inside the green circle. The planned obstacle-free trajectory to reach the reference is represented as a red line. The gripper autonomously detects the yolk while a Rapidly-exploring Random Tree motion planner computes, and continuously updates, an obstacle-free trajectory in order to reach it. Once the target is reached, the temperature is increased until the system classifies the gripper as closed. An obstacle-free trajectory then guides the gripper to the target where the temperature is decreased until the gripper is marked as open. Finally, the gripper is moved away from the drop-off area. Times (t) and temperatures (T) are shown on the bottom-left corner of each snapshot. *Please refer to the accompanying video for this experiment*

An extensive evaluation is performed to assess the capabilities of the systems and demonstrate the ability of grippers to manipulate biological material in unstructured environments, and to detect and sort micro-scale objects. In the first experiment, one static and two dynamic virtual obstacles are avoided by the gripper that is at the same time performing pick-and-place of a piece of egg yolk (Fig. 4). The dynamic obstacles are two circles of 0.5 mm diameter, while the static obstacle is a wall with length of 4 mm. The dynamic obstacles are modeled as first order linear holonomic systems. Their initial motion directions and speeds are reported in Fig. 4b. To simulate the tracking errors, we add zero-mean white Gaussian noise at a standard deviation *σ*=0.5 mm to the *x*− and *y*− coordinates of the dynamic obstacles. The noisy measured positions of the obstacles are provided to the motion planner in order to compute obstacle free paths. *Please refer to the accompanying video showing an example of the experiments.* Egg yolk was chosen due to its high content of cholesterol and fat content (58 % of its dry weight), representative of materials that are often manipulated in MIS interventions. To increase the resemblance between the experimental scenario and operations in biological environments, the tasks are performed on a base of porcine tissue. Based on a total of five trials, this experiment is completed with an average error on the drop-off of the object of 0.62 ±0.22 mm. The results were achieved at an average velocity of 1.81 ±0.71 mm/s for loaded grippers and 2.08 ±0.39 mm/s for unloaded grippers.

**Fig. 5:**
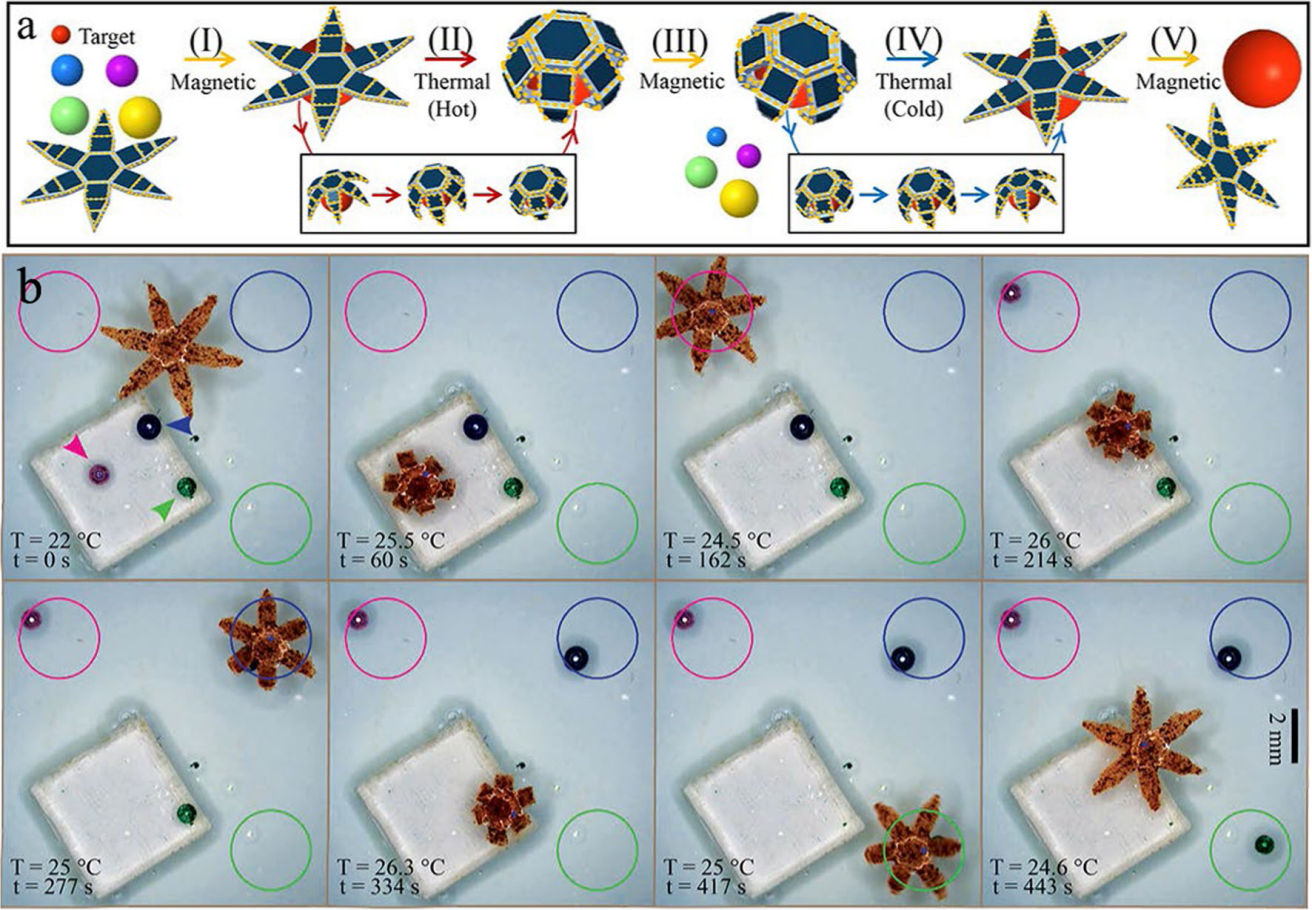
**a** Schematic of a representative autonomous sorting experiment. **b** Video snapshots that show the gripper autonomously detecting and sorting differently colored beads, pick-and-placing them in the respectively colored drop areas. The gripper autonomously classifies the micro-scale objects, and estimates the configuration of the grippers from Fourier descriptors to determine if the desired configuration is reached. Times (t) and temperatures (T) are shown on the bottom-left corner of each snapshot. *Please refer to the accompanying video for this experiment*

The second experiment tests if the grippers can autonomously recognize various objects and sort them.We perform an experiment in which the grippers recognize colored micro-scale objects and, one by one, pick-and-place them in the area marked with the corresponding color (Fig. 5). The manipulated objects are three spherical polyester beads of 0.5 mm diameter and 0.6 ±0.1 mg weight. A color segmentation algorithm similar to that described in Section 3 is used to extract the position of the three objects. *Please refer to the accompanying video showing an example of the experiments.* A total of five trials are conducted with a velocity and error on the drop-off of the micro-scale object error of 1.21 ±0.68 mm/s and 0.85 ±0.41 mm respectively. The average error of the micro-scale object always lays within the bounds of the radius of the drop-off location (1.5 mm).

## Conclusions

The paper has demonstrated the capabilities of soft grippers to autonomously perform micromanipulation tasks. To further validate the proposed approach, we have performed tests on animal tissue in unstructured environments that contain time-varying obstacles. All the experiments were directed by the decision-making, path-planning, and closed-loop control algorithms with no human intervention. Results show the ability of grippers to autonomously detect and adapt their behavior in response to changes in the surrounding environment. The demonstrated capabilities suggest that such grippers have the potential to enable delicate and precise manipulation in a broad variety of tasks, including microassembly, assisted fertilization and MIS. Moreover, due to their fully autonomous capabilities, they could find future applications in highly automatized processes as the population of PCB or the assembly of MEMS.

Future work will investigate the use of smaller scale grippers to offer more maneuverability and access to smaller environments. Also, 3D motion as well as operation in presence of fluid flow will be investigated. Operation in swarms will be studied to allow the parallelization of the micromanipulation tasks. Finally, we will exploit the metallic doped nature of these grippers in order to investigate the use of ultrasound or other clinically compatible imaging systems [41, 42].
